# Prolonged and intensive medication use are associated with the obesity paradox after percutaneous coronary intervention: a systematic review and meta-analysis of 12 studies

**DOI:** 10.1186/s12872-016-0310-7

**Published:** 2016-06-06

**Authors:** Xiao-Feng Tan, Jia-Xin Shi, and Meng-Hua Chen

**Affiliations:** Institute of Cardiovascular Diseases, the First Affiliated Hospital of Guangxi Medical University, Nanning, Guangxi 530027 People’s Republic of China

**Keywords:** Obesity paradox, Intensive medication, Mortality, Percutaneous coronary intervention

## Abstract

**Background:**

Obesity paradox is defined as the unexpected decrease in the total number of death which has been observed among patients who are overweight and obese compared to patients with normal weight after undergoing revascularization by percutaneous coronary intervention (PCI). Despite of so many recent studies which showed the existence of this phenomenon, prolonged and intensive medication use were only suggested to be among the reasons responsible for this ‘obesity paradox’ but it was never confirmed whether this hypothesis should really be considered true or not. Therefore, this study aimed to investigate whether prolonged and intensive medication use were associated with this obesity paradox after PCI.

**Methods:**

Medline, PubMed, EMBASE and the Cochrane Library were searched for studies showing the existence of this ‘obesity paradox’ in patients who underwent coronary revascularization by PCI and only articles comprising of medication use among the patients analyzed were considered relevant for this research. Medication use among the different subgroups of patients was calculated. Mortality was considered as the clinical endpoint in this study. Risk Ratio (RR) with 95 % Confidence Interval (CI) was used to express the pooled effect on discontinuous variables and the pooled analyses were performed with RevMan 5**.**3.

**Results:**

Twelve studies consisting of a total number of 91,582 patients was included in this meta-analysis. An intensive medication use after the hospital discharge and during the follow up period after PCI was observed in the subgroup of obese patients, followed by the overweight patients and the normal weight patients respectively. Our results showed that the short-term (30 days) mortality in overweight and obese patients was significantly lower compared to the normal weight patients with RR: 0.72; 95 % CI: 0.56-0.92, *p =* 0.008 and RR: 0.47, 95 % CI: 0.34-0.65; *p <* 0.00001 respectively. The long-term (≥ one year) mortality was also significantly lower in the overweight and the obese groups with RR: 0.74, 95 % CI: 0.67-0.82; *p <* 0.00001 and RR: 0.63, 95 % CI: 0.55-0.72; *p <* 0.00001 respectively.

**Conclusion:**

Our study has confirmed to some extent, that prolonged and intensive use of medications which were more prominent in patients who were overweight and obese during the follow up period, might apparently be among the reasons responsible for this obesity paradox after PCI.

## Background

Obesity paradox is referred to as the unexpected decrease in the total number of deaths observed among patients who are overweight and obese, compared to normal weight patients after undergoing revascularization by percutaneous coronary intervention (PCI) [1–3]. In contrast to patients with normal weight, because overweight and obese patients are candidates with high risk for cardiovascular diseases, and most probably suffer from co-morbidities such as hypertension, dyslipidemia, metabolic syndrome and diabetes mellitus at an early age, prolonged and intensive medical treatment including anti-hypertensive drugs such as diuretics and angiotensin converting enzyme inhibitors (ACEI), lipid-lowering agents such as statin, oral anti-hyperglycemic agents and anti-platelet medications such as aspirin to prevent and treat their underlying clinical conditions, eventually combined with daily exercises, change in diet, and intentional weight loss included in their treatment regimens are strictly considered at an early age.

Studies have shown that even though these patients have a high risk profile at the time of PCI, the use of multiple cardiovascular medications was associated with favorable clinical outcomes [4]. Certain studies have mentioned that the difference in optimal use of medications at follow-up among the subgroups with a high body mass index (BMI) when compared to patients with normal weight, has also partly contributed to this obesity paradox [5–7].

Despite of so many recent studies which showed the existence of this phenomenon, prolonged and intensive medication use were only suggested to be among the reasons which were responsible for this ‘obesity paradox’ but it was never confirmed whether this hypothesis should really be considered true or not [8]. Therefore, this study aimed to investigate whether prolonged and intensive medication use were associated with this obesity paradox after PCI.

## Methods

### Search strategy

Studies showing the existence of this ‘obesity paradox’ in patients who underwent coronary revascularization by PCI were searched through Medline, PubMed, EMBASE and the Cochrane Library by typing the words and phrases ‘obesity paradox and percutaneous coronary intervention’, and ‘obese and mortality and percutaneous coronary intervention’. To further enhance this search, the term ‘overweight and obese’ which was interchangeable with the term ‘high BMI’ was also used. Moreover, the abbreviation ‘PCI’ was used and it was also replaced by the word ‘coronary angioplasty’. Only studies published in English were considered in this search process. Table 1 shows the number of citations obtained during the search of studies by using appropriate keywords.

**Table 1:** Number of articles obtained during the search process

Phrase or words used during the search strategy	No of articles obtained (n)
obesity paradox and percutaneous coronary intervention	1098
obese and mortality and percutaneous coronary intervention	1312
overweight and obese/high BMI and PCI	345
Obesity paradox and PCI	809
Total no of articles obtained (n)	3564

### Inclusion and exclusion criteria

Studies were included if:

- They were randomized controlled trials (RCTs) or observational studies comparing normal weight, overweight and obese patients after revascularization with PCI.
- They reported mortality as their clinical endpoints.
- They also included the medications prescribed at discharge or during the follow-up period after PCI. This feature was a major part in assessing studies which were eligible for this research.

Studies were excluded if:

- They were neither RCTs nor observational studies (they were meta-analyses and case studies).
- They did not compare overweight and obese patients with normal weight patients after PCI.
- Mortality was not reported among their clinical end points.
- Medication use or prescription at discharge or follow up was not mentioned (studies were not included if mortality was among the endpoints but medication prescribed at discharge or follow-up was not mentioned).
- Only their abstracts were made available.
- Data for the control group (normal weight patients) were not available.

### Outcomes and follow up

In hospital, short-term (30 days to one year) and long-term mortality (≥1 year) were considered as the only outcome for this study.

### Definitions

- **Normal weight patients:** were defined as patients with a BMI > 18.5 but <25 kg/m^2^.
- **Overweight patients:** were defined as patients with a BMI > 25 kg/m^2^ but <30 kg/m^2^.
- **Obese patients:** were defined as patients with a BMI > 30 kg/m^2^.
- **High BMI patients**: including both overweight and obese patients, were defined as patients with a BMI > 25 kg/m^2^.

Exceptions were also present in this study. Among all the studies included, one study assumed normal weight patients to have a BMI > 18.5 to <23 kg/m^2^ whereas another study assumed normal weight patients to have a BMI of 18.5- <24 kg/m^2^ instead of the standard BMI range of 18.5 to 25 kg/m^2^. In this same study, BMI of 23–27.5 kg/m^2^ and >27.5 kg/m^2^ were considered for overweight and obese patients respectively. Another study assumed a BMI of 24-27 kg/m^2^ and >27 kg/m^2^ to be overweight and obese respectively.

**Medication use:** included multiple cardiovascular medications such as beta-blockers, angiotensin converting enzyme inhibitors (ACEI) or angiotensin receptor blockers (ARB), lipid-lowering medications (statins), diuretics and antiplatelet drugs such as aspirin and clopidogrel used earlier before PCI, or prescribed at discharge or during the follow-up period to be used continuously in order to prevent complications.

**Prolonged medication use**: defined as the use of these cardiovascular medications since the beginning of previously diagnosed conditions such as hypertension or hypercholesterolemia/hyperlipidemia in these high risk profile patients, which could be earlier before PCI or during the follow-up period after PCI. For example, beta blockers used to treat hypertension and statin used earlier for the prevention or treatment of hyperlipidemia in these overweight and obese patients, and later these drugs were continued to be used together along with additional medications for example clopidogrel use for one year and lifelong aspirin post PCI or the continuous use of statin during the follow up period.

**Intensive medication use and treatment:** We believe that both the mg/kg and the one-size-fits-all strategy for prescribing medications are outdated as individual body size and composition characteristics of patients are likely to clinically significantly affect pharmacokinetic parameters. Further adjusting the dosage could improve efficacy in these high BMI patients. Hence, a higher drug dosage use calculated according to the body weight will vary depending on the size/weight of the individual. Total body weight, ideal or adjusted body weights are used for these high BMI patients. Use of medications for the prevention of unwanted conditions beforehand such as statin use even if the patient does not have hyperlipidemia yet but is at high risk for this condition, frequent home glucose monitoring in those suffering from diabetes, continuous visit to the physician for medical check-ups and counseling including lifestyle counseling, weight management and other health tips were all considered in this intensive treatment plan.

- **Mortality:** was defined as death of all causes including cardiac and non-cardiac deaths.
- **In-hospital mortality:** was defined as death occurring during the hospital stay just after PCI but before being discharged from the hospital.
- **Short-term mortality:** was defined as death occurring after hospital discharge and during a follow up period of one year after PCI. This short-term follow up period was divided into a follow up period of 30 days and a follow up period during one year. Follow up period at one year has been classified in the long-term category.
- **Long-term mortality**: was defined as death occurring at one year or after one year (≥1 year).

### Data extraction process

The authors X.F.T. and J.X.S. independently reviewed all the studies and assessed them for eligibility. Several information was extracted including the characteristics/features of the patients involved, the total number of normal weight, overweight and obese patients, the medication used in each subgroup of patients, the mortality reported as well as the follow up periods. Selected studies were carefully checked and filtered for the inclusion of medication use at discharge or during the follow-up period. Any disagreement raised during this data extraction process has been carefully discussed between these two authors, and if they could not reach a decision, M.H.C., the third author was called to discuss and resolved the problem. Assessment of the bias risks within the studies with the components recommended by the Cochrane Collaboration [9] was not conducted since all the studies were observational cohorts.

### Statistical analysis

A bar chart has been used to represent the percentage of patients using specific medications among the different BMI subgroups and Risk Ratio (RR) with 95 % confidence interval (CI) was used to express the pooled effect on discontinuous variables related to post-PCI mortality. Heterogeneity across trials was assessed using the Cochrane Q-statistic (*p* 0 **·** 05 was considered significant whereas *p >* 0.05 was considered statistically insignificant) and the I^2^-statistic which described the percentage of total variation across studies; that is, due to heterogeneity rather than chance (whereby an I^2^ with low percentage represented a lower heterogeneity whereas an increasing percentage denoted an increasing heterogeneity). If I^2^ was less than 50 %, a fixed effect model was used. However, if I^2^ was more than 50 %, a random effect model was used. Publication bias was visually estimated by assessing funnel plots. We calculated RR and 95 % CIs for categorical variables. The pooled analyses were performed with RevMan 5**.**3 software.

## Results

**Fig. 1:**
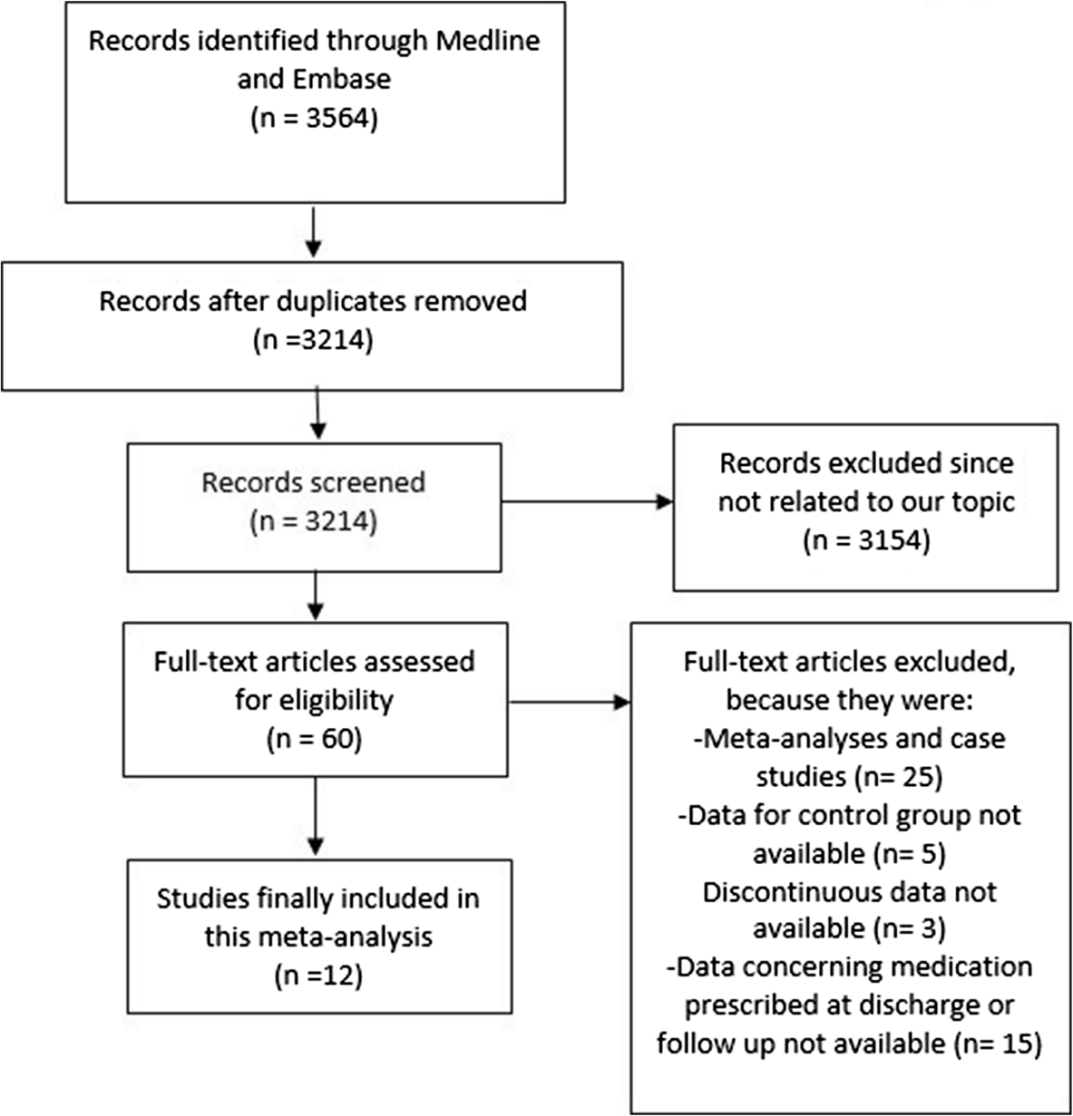
Flow diagram for the study selection

The preferred reporting items for systematic reviews and meta-analyses (PRISMA) guideline was followed in this study. During our search for eligible studies, based upon titles and abstracts, 3,564 articles were obtained from the above mentioned electronic databases. 3,504 publications have been eliminated on the basis of title and abstract because they were not related to our topic or they were duplicates. 60 full-text articles were assessed for eligibility. Further articles were excluded since they were: meta-analyses or case studies, data for the control group were not available, and data concerning medications prescribed at discharge or during the follow up period were not reported. Finally, 12 studies with a total number of 91,582 patients satisfied all the inclusion criteria and were confirmed for this meta-analysis. The flow diagram for identifying potentially eligible studies and the reasons for inclusion and exclusion have been represented in Fig. 1.

The baseline characteristics of the studies included in this meta-analysis have been listed in Table 2.

**Table 2:** shows the baseline characteristics of the included studies

Study	Patients (n)	Age (yrs)	Men (%)	HT (%)	Ds (%)	CS (%)	DM (%)
	N/Ov/Ob	N/Ov/Ob	N/Ov/Ob	N/Ov/Ob	N/Ov/Ob	N/Ov/Ob	N/Ov/Ob
Das2011 [10]	11780/19391/18158	66.0/60.0/56.3	61.9/75.3/69.6	56.9/58.5/69.3	43.4/49.7/55.4	45.0/43.1/42.1	14.8/19.5/35.3
Kang 2010 [11]	1253/1959/483	64.7/59.4/56.5	70.9/79.5/76.4	39.9/47.2/54.2	7.0/10.4/12.7	60.3/64.2/66.2	22.5/23.5/28.7
Lancefield 2010 [8]	1189/2016/1426	67.4/64.7/61.4	73.2/79.0/68.8	58.6/61.9/74.1	68.5/71.7/75.6	23.4/19.9/23.2	15.9/22.3/33.6
Akin 2012 [12]	1436/2839/1531	66.1/65.5/63.7	69.8/78.5/72.1	75.4/84.2/91.2	75.9/81.0/83.5	26.3/21.4/20.4	21.5/29.2/46.9
Cheng 2013 [13]	477/428/340	67.7/61.9/57.9	80.1/84.3/85.0	53.5/55.8/56.2	NM	44.0/40.4/36.5	36.7/36.4/32.9
He 2015 [14]	489/447/81	NM	67.7/68.0/58.0	62.8/76.3/72.8	48.7/51.0/50.6	16.4/13.6/14.8	27.4/26.4/24.7
Wang 2012 [15]	1592/3026/1465	60.9/58.8/56.8	62.7/64.7/66.4	58.0/60.8/70.9	30.3/30.9/38.1	44.4/42.4/40.2	34.6/33.5/38.4
Numasawa 2015 [16]	5945/3100/635	69.4/65.4/59.2	79.3/83.5/78.7	70.9/80.0/84.9	63.9/72.9/78.4	33.3/38.7/44.7	40.0/46.4/58.6
Buettner 2007 [17]	551/824/292	65.9/64.7/62.7	67.0/75.0/61.0	55.0/62.0/70.0	60.0/68.0/69.0	21.0/25.0/24.0	17.0/18.0/25.0
Kaneko2013 [18]	640/417/56	66.8/63.2/55.2	81.4/87.8/92.9	61.3/71.7/69.6	56.6/66.7/71.4	33.4/41.7/62.5	33.4/35.7/48.2
Schenkeveld 2012 [5]	2095/2956/1246	62.0/62.0/59.0	70.0/77.0/67.0	34.0/39.0/55.0	73.0/76.0/79.0	27.0/22.0/23.0	12.0/16.0/27.0
Younge2013 [19]	354/468/197	63.0/63.0/60.0	70.0/76.0/68.0	32.0/28.0/25.0	56.0/59.0/61.0	35.0/42.0/54.0	10.0/16.0/24.0

Abbreviations: *N* normal weight, *Ov* overweight, *Ob* obese, *NM* not mentioned, *HT* hypertension, *Ds* dyslipidemia, *CS* current smoker, *DM* diabetes mellitus, *yrs* years

According to the baseline features, the study published by Das et al. [10] involved the majority of patients in all the different subgroups of patients (11,780 patients who had a normal weight, 19,391 patients who were overweight and 18,158 patients who were obese). Obese patients were younger than patients with normal weight and male patients were higher in all the BMI subgroups compared to female patients. The study by Akin et al. [12] had the highest number of obese patients with hypertension. Moreover, the study by Kang et al. [11] had the highest number of patients who were current smokers. Most of the patients who were obese also had diabetes mellitus.

The medications prescribed at discharge and during the follow up period have been represented in Table 3.

**Table 3:** shows the medications at discharge and during follow-up used by the patients in the different BMI subgroups

Discharged and follow-up Medications	Normal weight patients	Overweight patients	Obese patients
Aspirin (%)	94.7	95.1	95.0
Clopidogrel (%)	82.4	83.1	83.2
Statin (%)	76.8	79.3	81.3
ACEI/ARBs (%)	55.8	65.2	68.8
Beta blocker (%)	70.4	72.1	73.8

Abbreviations: *ACEI* angiotensin converting enzyme inhibitor, *ARB* angiotensin receptor blocker

**Fig. 2:**
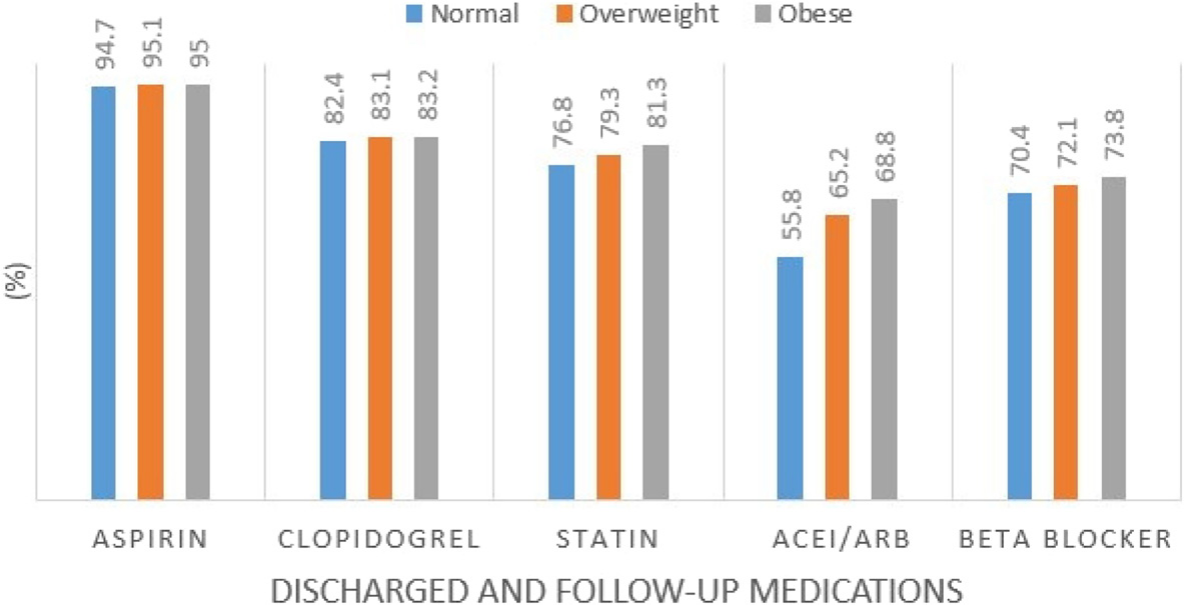
Medications at discharge and during the follow-up period

According to Table 2, 94.7 % of patients with normal weight, 95.1 % of the patients who were overweight and 95.0 % of the patients who were obese used aspirin. 82.4 %, 83.1 % and 83.2 % of the patients with normal weight, overweight and obese respectively used clopidogrel. Normal weight, overweight and obese patients using beta blocker and statin were 70.4 % and 76.8 %, 72.1 % and 79.3, and 73.8 % and 93.3 % respectively. Medication use was higher among patients who were overweight and highest among patients who were obese. Overweight patients used more medications than normal weight patients whereas obese patients used more medications than overweight patients after PCI. A flow chart showing the percentage of medication use by the different subgroups of patients has been illustrated in Fig. 2.

The percentage of in-hospital death among the different subgroups of patients after PCI has been represented in Table 4. In-hospital death was lower in the overweight and obese patients compared to the normal weight patients with the exception of patients from the study Numasawa et al.[16].

**Table 4:** shows the Percentage of In-hospital Death among the different subgroups of patients

	Normal weight	Overweight	Obese
	(%)	(%)	(%)
Das 2011	7.4	4.7	4.4
Kang 2010	4.4	3.0	1.7
Lancefield 2010	1.2	0.9	0.5
Akin 2012	0.8	0.5	0.3
He 2015	1.0	0.4	0.0
Numasawa 2015	2.1	1.2	2.7

Short and long-term deaths in patients with normal weight, overweight and obese after PCI have been represented in Tables 5 and 6 respectively. Short and long-term deaths were lower among patients who were overweight and obese compared to those with normal weight with the exception of patients from the study published Wang et al.[15].

**Table 5:** shows the Percentage of Short-term Death among the different subgroups of patients

30 days follow up	Normal weight	Overweight	Obese
	(%)	(%)	(%)
Buettener 2007	2.0	2.3	1.0
Lancefield 2010	1.6	1.1	1.05
Kang 2010	1.2	1.0	0.2
Cheng 2013	19.3	12.1	8.5
30 days to 1 year follow up			
Kang 2010	2.0	1.6	0.9
Lancefield 2010	4.0	2.9	2.1

**Table 6:** shows the Percentage of Long-term (≥ one year) Death among the different subgroups of patients

	Normal weight	Overweight	Obese
	(%)	(%)	(%)
Kang 2010	3.3	2.6	1.1
Buettner 2007	7.4	5.1	2.7
Akin 2012	3.3	2.4	2.4
Younge 2013	7.1	6.3	3.0
Wang 2012	2.4	2.3	2.7
He 2015	1.8	1.8	0.0
Kaneko 2013	4.8	2.2	3.6
Schenkeveld 2012	21.2	15.0	13.0

### Results of the meta-analysis

#### In hospital mortality

**Fig. 3:**
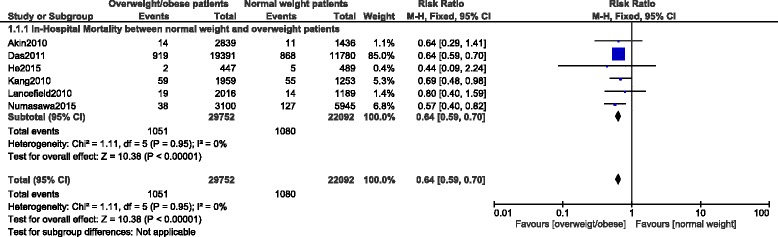
In-hospital mortality among overweight patients after PCI

**Fig. 4:**
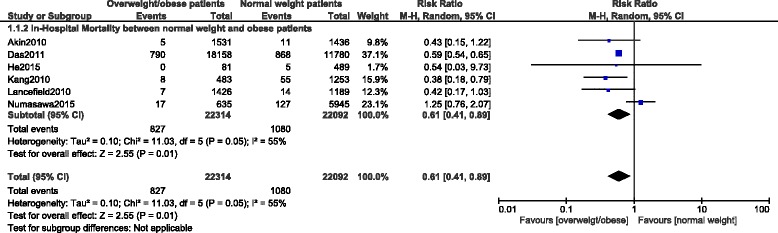
In-hospital mortality among obese patients after PCI

In-hospital mortality among overweight and obese patients were significantly lower compared to the normal weight patients with RR: 0.64, 95 % CI: 0.59-0.70; *p <* 0.00001 for patients who were overweight and with RR: 0.61, 95 % CI: 0.41-0.89; *p =* 0.01 for patients who were obese. These results have been shown in Figs. 3 and 4 respectively.

#### Short term mortality

Short term mortality was classified into two parts. One part compared the mortality during a 30-days period while the other part compared the mortality during a follow up period from 30 days to one year.

During the 30 days follow up, mortality was significantly lower in the overweight and obese groups with RR: 0.72, 95 % CI: 0.56–0.92; *p =* 0.008 and RR: 0.47, 95 % CI: 0.34–0.65; *p <* 0.00001 respectively.

**Fig. 5:**
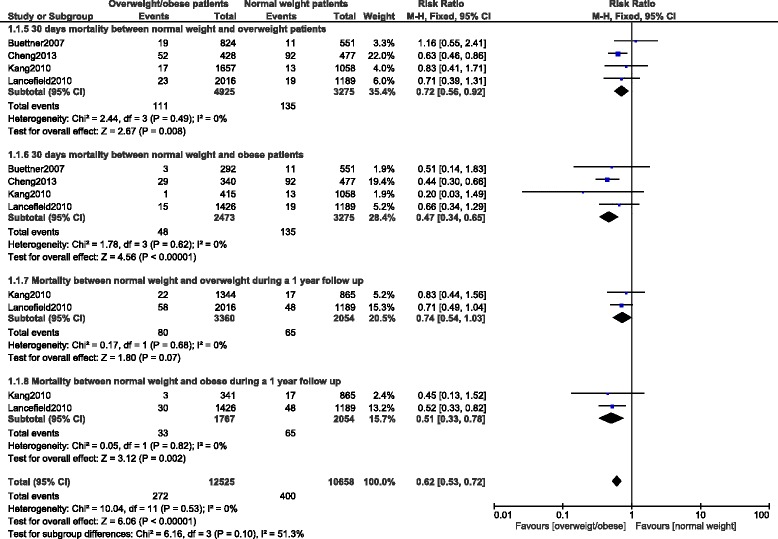
Short-term mortality after PCI

When a follow up from 30 days to one year was considered, mortality was still significantly lower in the obese group with RR: 0.51, 95 % CI: 0.33–0.78; *p =* 0.002. Results for the short term follow up have been illustrated in Fig. 5.

#### Long-term mortality

**Fig. 6:**
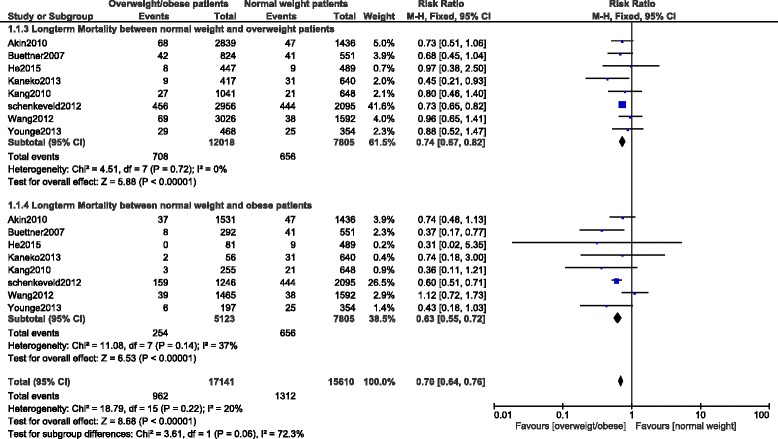
Long-term mortality after PCI

Long-term mortality was also significantly lower in the overweight and obese patients with RR: 0.74, 95 % CI: 0.67-0.82; *p <* 0.00001 and RR: 0.63, 95 % CI: 0.55–0.72; *p <* 0.00001 respectively. The result for the long-term follow up has been represented in Fig. 6.

**Fig. 7:**
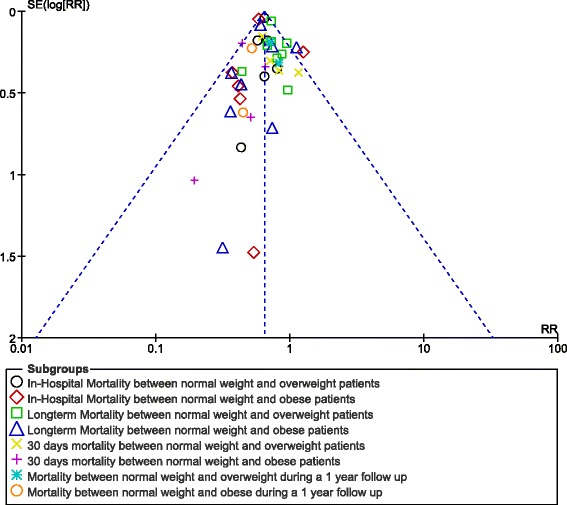
Funnel plot for the sensitivity analysis

For all of the above analyses, sensitivity analyses yielded consistent results. Based on a visual inspection of the funnel plot, there has only been little evidence of publication bias for the included studies that assessed mortality during different follow up periods. The funnel plot has been illustrated in Fig. 7.

## Discussion

Several hypotheses have previously been suggested for this obesity paradox. It was suggested in many studies that the prolonged and intensive use of medications in overweight and obese patients could be among the reasons responsible for this phenomenon [5, 8]. Overweight and obese patients are more exposed to cardiovascular risk factors and because majority of these patients suffer from conditions like hypertension, dyslipidemia and diabetes mellitus, they are first treated for these conditions since an early age [20, 21]. Increased use of medications such as beta blockers, statins and ACEI/ARB, with increased dosage, along with additional medications such as antiplatelet agents (aspirin and clopidogrel) prescribed after PCI seem to have all contributed to this paradox.

Our results showed that medication use was more intense among patients who were obese followed by patients who were overweight. Moreover, our results showed that since cardiac medications were intensively used among these high risk patients, these medications could be associated with this significant decrease in in-hospital, short term as well as the long-term mortality when compared to patients with normal weight who did not use intensive medications.

In our study, we tried to investigate the impact of prolonged and intensive medication use on the obesity paradox and our results could partly show the association of intensive medication use with this phenomenon. However, even in other studies, it was suggested that intensive medication use among patients with high BMI was associated with a lower mortality risk. Lancefield et al. concluded that patients who were obese had a higher rate of guideline-based medication use at 12 months, which might in part explain the obesity paradox seen after PCI [8]. Steinberg et al. also found that increased BMI was related to an increased use of guideline-based medical therapy both in-hospital and at discharge in 130,139 patients hospitalized for coronary artery disease [22].

Moreover, in the study published by Kennedy et al., the authors found that BMI was associated with an increased mortality risk among obese patients who were not receiving beta-blocker or renin angiotensin system-blockade (RAS) [23]. However, non-pharmacological measures such as smoking cessation, cardiac rehabilitation, and dietary counseling have also shown to be more prominent and visible in patients who were overweight and obese altogether contributing to this obesity paradox [24].

Schenkeveld et al. also supported the fact that patients with high BMI were given priority on optimal medical treatment, which could in a certain way explain the decrease in mortality rate after PCI thus contributing to this obesity paradox [5]. Their study found that this inverse relationship between high BMI and mortality rate persisted even during the long-term follow-up after PCI. Patients from the overweight and obese groups showed almost 30 % lower mortality rate compared to patients with a normal BMI. In their cohort study, patients with a high BMI were often prescribed beta blocker and ACEI enormously when compared with normal weight patients indirectly explaining this obesity paradox.

In addition, the study published by Claudio et al. found that thirty-day readmissions related to ‘acute coronary syndrome (ACS)/heart failure’ admission diagnosis were rare among patients with high BMI. But only those patients with ‘ACS/heart failure’ who had periprocedural myocardial infarction as the only independent predictor showed a detrimental impact on prognosis [25]. In their study, periprocedural myocardial infarctions was a marker for a higher risk of atherothrombotic events and their detrimental effect on ejection fraction, potentially leading to hemodynamic instability. During the early period of discharge, these patients were closely monitored and medications were administered to their maximal tolerated dosage. This result was consistent with our study in which showed intensive use of medications during the follow up period might be among the reasons responsible for a lower mortality risk among patients with high BMI thus contributing to this obesity paradox. However, not only prolonged and intensive medication use could be associated with this phenomenon. The beneficial effect of intensive medical therapy among patients with high BMI might also have been influenced by a change in lifestyle. Other factors such as healthy dieting, regular exercises, quitting smoking and referral to cardiac rehabilitation as mentioned above could also have contributed to this beneficial long-term prognosis in these patients [5]. In addition, as shown in the baseline features of this study, a younger age among patients with high BMI could also be a factor contributing to this ‘obesity paradox’. Certain studies have also suggested larger coronary arteries among patients who are overweight and obese could also be associated to this lower mortality risk after PCI as mentioned in the study by Bundhun et al. [26]. Prolonged and intensive medication use might not be the only factor contributing to this obesity paradox after PCI.

This study is new in the way that it is among the first study investigating whether prolonged and intensive medication use are associated with the obesity paradox after PCI. Several studies have shown the existence of this phenomenon but none has previously investigated its association with the use of aggressive medications.

### Limitation

This study also has limitations. First of all, extremely obese patients were also included in the category of obese patients. This could possibly have an effect on the result since obesity could further be divided into several classes. Secondly, one or two studies included in our meta-analysis assumed normal weight patients to have a BMI > 20 but <25 kg/m^2^ whereas other studies classified overweight patients to have a BMI ranging from 24 kg/m^2^ to <27 kg/m^2^ while obese patients had a BMI of >27 kg/m^2^ instead of >30 kg/m^2^. This could have an effect on our results. In addition, our study did not consider some factors which such as the clinical presentation (acute coronary syndrome vs stable angina) and procedural features including lesion type, number of implanted stents, type of the stents which could all have an influence on the prognosis of patients after PCI. Another limitation for this study might be considered to the fact that the in-hospital follow up was too short for the medications to be effective. So, the intensive use of medications should be highly associated with the long-term mortality risk in these patients after PCI.

## Conclusion

Our study has confirmed to some extent, that prolonged and intensive use of medications which were more prominent in patients who were overweight and obese during the follow up period, might apparently be among the reasons responsible for this obesity paradox after PCI.

Further investigations are required to completely solve this issue.

## Abbreviations

ACEI, angiotensin-converting enzyme inhibitor; ARB, angiotensin receptor blocker; BMI, body mass index; PCI, percutaneous coronary intervention; RCTs, randomized controlled trials.
